# Electrophysiological Activity of Multifunctional and Behaviorally Specialized Spinal Neurons Involved in Swimming, Scratching, and Flexion Reflex in Turtles

**DOI:** 10.1523/ENEURO.0038-24.2024

**Published:** 2024-07-19

**Authors:** Madison M. Morris, Zhao-Zhe Hao (郝赵哲), Ari Berkowitz

**Affiliations:** School of Biological Sciences, University of Oklahoma, Norman, Oklahoma 73019

**Keywords:** central pattern generator, flexion reflex, interneuron, locomotion, scratching, spinal cord

## Abstract

The adult turtle spinal cord can generate multiple kinds of limb movements, including swimming, three forms of scratching, and limb withdrawal (flexion reflex), even without brain input and sensory feedback. There are many multifunctional spinal neurons, activated during multiple motor patterns, and some behaviorally specialized neurons, activated during only one. How do multifunctional and behaviorally specialized neurons each contribute to motor output? We analyzed in vivo intracellular recordings of multifunctional and specialized neurons. Neurons tended to spike in the same phase of the hip-flexor (HF) activity cycle during swimming and scratching, though one preferred opposite phases. During both swimming and scratching, a larger fraction of multifunctional neurons than specialized neurons were highly rhythmic. One group of multifunctional neurons was active during the HF-on phase and another during the HF-off phase. Thus, HF–extensor alternation may be generated by a subset of multifunctional spinal neurons during both swimming and scratching. Scratch-specialized neurons and flexion reflex-selective neurons may instead trigger their respective motor patterns, by biasing activity of multifunctional neurons. In phase-averaged membrane potentials of multifunctional neurons, trough phases were more highly correlated between swimming and scratching than peak phases, suggesting that rhythmic inhibition plays a greater role than rhythmic excitation. We also provide the first intracellular recording of a turtle swim-specialized neuron: tonically excited during swimming but inactive during scratching and flexion reflex. It displayed an excitatory postsynaptic potential following each swim-evoking electrical stimulus and thus may be an intermediary between reticulospinal axons and the swimming CPG they activate.

## Graphical abstract

**Figure:**
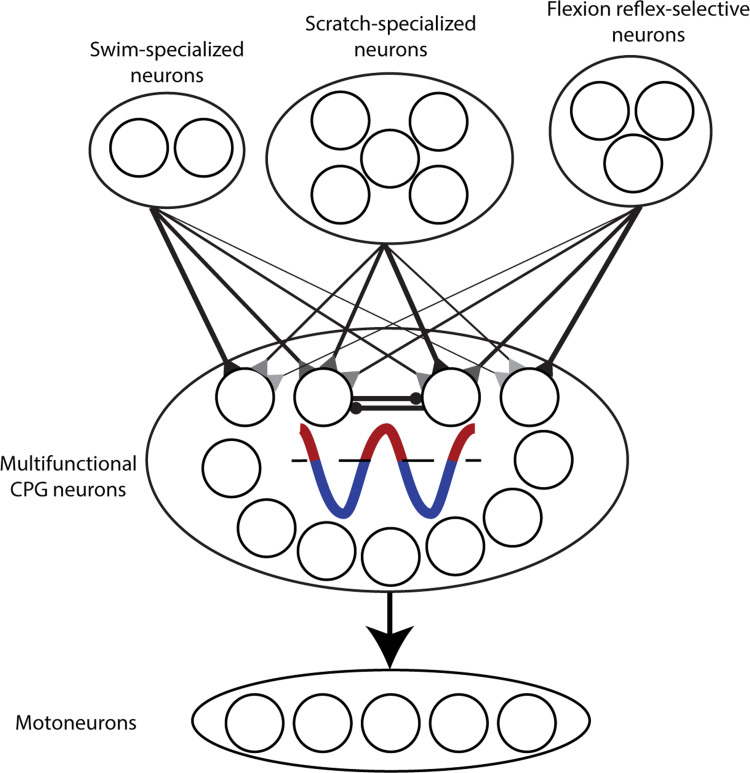
Visual Overview

## Significance Statement

We analyzed in vivo intracellular recordings of multifunctional and behaviorally specialized turtle spinal neurons, including scratch-specialized and flexion reflex-selective neurons. During both swimming and scratching motor patterns, there were more highly rhythmic multifunctional neurons than behaviorally specialized neurons; their rhythmic modulation appeared to be caused mostly by inhibition. Multifunctional neurons may form core elements of central pattern generators, while behaviorally specialized neurons trigger each motor pattern. We also recorded intracellularly the first turtle swim-specialized neuron.

## Introduction

Central pattern generators (CPGs) are networks of central nervous system neurons that produce basic patterns of motoneuron activity underlying many fundamental movements, especially rhythmic movements like breathing, chewing, and locomotion (Marder and Calabrese, 1996; Arshavsky et al., 2016). The same muscles and motoneurons are typically used for multiple kinds of movements. How do CPGs cause motoneurons to be activated differently to produce the appropriate movement for each circumstance? In small, invertebrate nervous systems, many CPG neurons are multifunctional—they contribute to more than one type of movement—with sensory stimulation and/or neuromodulation altering CPG function and motor output (Morton and Chiel, 1994; Briggman and Kristan, 2008). Sometimes, however, CPGs for different movements involving the same muscles and motoneurons are completely separate or dedicated (Ramirez and Pearson's, 1988; Hennig, 1990).

The vertebrate situation is less clear, due to greater complexity and technical challenges (Sengupta and Bagnall, 2023), but most vertebrate networks also appear to be multifunctional (Briggman and Kristan, 2008). The spinal cord in hatchling tadpoles and larval zebrafish uses several types of multifunctional CPG neurons to generate different axial body movements—swimming, struggling, and escape—but also has some behaviorally specialized neurons (Berkowitz et al., 2010). The control of limb movements in adults is more complex and less well understood. Studies of spinal cord selection and generation of limb movements in adults have largely used turtles, which can produce the motor patterns underlying swimming, three forms of scratching, and limb withdrawal (flexion reflex) without brain input and movement-related sensory feedback (Berkowitz and Hao, 2011; Stein, 2018; Berg, 2020) and are unusually resistant to hypoxia (Lutz and Milton, 2004; Cox and Gillis, 2020; Fago, 2022; Lefevre and Nilsson, 2023).

Two kinds of evidence suggest that the CPGs for turtle swimming, scratching, and flexion reflex share key timing elements. First, two stimuli that individually trigger two distinct motor patterns when delivered together can produce motor pattern blends, called hybrids and switches; also, a brief stimulus of one type can alter the timing of subsequent cycles of an ongoing motor pattern of another type (Stein et al., 1986; Currie and Stein, 1989; Juranek and Currie, 2000; Berkowitz and Hao, 2011; Hao et al., 2011; Elson and Berkowitz, 2016; Hao and Berkowitz, 2017). Second, single-neuron recordings have shown that many spinal interneurons (i.e., spinal neurons that are neither sensory neurons nor motoneurons) are multifunctional, defined as increasing their spike rate during both swimming and scratching; many are also activated during flexion reflex (Berkowitz, 2002, 2008). However, we have also discovered two sets of behaviorally specialized spinal interneurons: scratch-specialized neurons, which are activated during one or more forms of scratching, but not activated and often inhibited during swimming, and flexion reflex-selective neurons, which are activated at short latency during flexion reflex, but not activated and often inhibited during swimming and scratching (Berkowitz, 2002, 2007, 2008; Bannatyne et al., 2020).

How do multifunctional and behaviorally specialized spinal interneurons work together to produce the right limb movement at the right time? Earlier studies relied on extracellular single-neuron recording (Berkowitz, 2002), which provides no information on subthreshold membrane potentials. Later studies used intracellular recording combined with dye injection to examine electrophysiological, morphological, and immunocytochemical properties of these interneurons, including their neurotransmitter and whether or not their axons contact motoneurons directly (Berkowitz, 2005, 2007, 2008; Berkowitz et al., 2006; Bannatyne et al., 2020). Due to the requirement to have both electrophysiology and morphology of these neurons, however, the usable datasets were relatively small.

Here, we studied in detail the in vivo intracellular electrophysiology of multifunctional interneurons and each type of behaviorally specialized interneurons using a larger dataset. We find that more multifunctional neurons than behaviorally specialized neurons are highly rhythmic; their rhythmic modulation appears to be created mostly by inhibition. Thus, inhibitory multifunctional neurons may form the core CPG for both swimming and scratching, while behaviorally specialized neurons may each bias the multifunctional CPG in a particular direction. We also provide intracellular recordings of a swim-specialized neuron for the first time in turtles.

## Materials and Methods

### Dataset

The recordings analyzed in this study were obtained from experiments conducted in 2011–2018 for a project combining intracellular electrophysiology with morphology and immunocytochemistry (Bannatyne et al., 2020). The neurons analyzed here did not have adequate morphological data to be included in the previous study and have not been analyzed or reported on until now.

### Animal preparation

Adult red-eared sliders (*Trachemys scripta elegans*, 360–850 g, both sexes, *n* = 44) were placed in ice for ≥2 h to induce hypothermic analgesia prior to three surgical procedures, which were performed with the turtle in ice. First, the spinal cord was transected between Dorsal Segments 2 and 3 (D2–D3). Second, D6–D10 and Sacral Segments 1 and 2 (S1–S2) were exposed; D8–D10 and S1–S2 are the five segments of the hindlimb enlargement. Third, several right hindlimb motor nerves were separated from other tissues to prepare them for recording: the hip flexor, ventral puboischiofemoralis internus, pars anteroventralis (HF); the hip extensor, flexor cruris, pars flexor tibialis internus (HE); and one or more of the knee extensors, triceps femoralis, pars iliotibialis (IT), pars ambiens (AM), and/or pars femorotibialis (FT). Gallamine triethiodide (8 mg/kg; Sigma-Aldrich) was injected intramuscularly to immobilize the animal, which was then artificially ventilated (Harvard Apparatus) for the duration of the experiment. These procedures have been described in more detail previously (Berkowitz, 2001b). The Institutional Animal Care and Use Committee of the University of Oklahoma approved all procedures.

### Motor pattern stimulation

Nerve recordings were obtained by pairs of 100 μM silver wires (California Fine Wire) wrapped around each nerve, suspended in a pool of mineral oil within a wax well, amplified, and filtered (x1,000; bandpass 0.1–1.0 kHz; A-M Systems or Grass/Natus Neurology). Fictive forward swimming (henceforth, just “swimming”) was evoked by electrical stimulation of the contralateral D3 lateral funiculus (0.1 ms, 200–400 μA pulses at 40 Hz; AMPI). Fictive scratching (henceforth, just “scratching”) was evoked by mechanical stimulation using a glass probe with a fire-polished tip in each scratch form's receptive field. Fictive flexion reflex (henceforth, just “flexion reflex”) was elicited by a tap to the dorsum of the foot, with the same glass probe used for scratching, or by electrical stimulation to the skin of the dorsal foot (five 1-ms pulses at 100 Hz). Motor patterns were evoked in a pseudorandom order.

### Intracellular recordings

Intracellular recordings were obtained using sharp microelectrodes fabricated with a P-97 puller (Sutter Instrument) from 1.0 mm outer-diameter/0.78 mm inner-diameter borosilicate glass with filament (Sutter Instrument) and filled with either 0.5 M KCl (Thermo Fisher Scientific) or 5% NaCl (Thermo Fisher Scientific), pH 7.0, in addition to dyes. Microelectrodes were slightly beveled (BV-10, Sutter Instrument) to resistances of 30–120 MΩ, inserted into the right side of one of the five segments of the hindlimb enlargement (D8, D9, D10, S1, and S2) or the segment just rostral to the hindlimb enlargement (D7) and advanced via a Burleigh Inchworm piezoelectric microdrive (Burleigh/EXFO). All neurons were recorded ≤1,000 µm from the dorsal surface to largely exclude motoneurons (Berkowitz and Stein, 1994a; Berkowitz, 2001b). Recordings were obtained via an Intra 767 amplifier (World Precision Instruments). Nerve and intracellular recordings were stored in a digital audio tape recorder (TEAC America) and/or a PC (Micro1401 hardware and Spike2 software, Cambridge Electronic Design). Each neuron had to have clear postsynaptic potentials, ≥30 mV action potentials, and be recorded during ≥2 cycles of swimming and ≥2 cycles of scratching (rostral, pocket, and/or caudal) to be included in this study. We defined multifunctional and specialized neurons as previously (Berkowitz, 2002): multifunctional neurons increase their mean spike rate during both swimming and scratching; scratch-specialized neurons increase their mean spike rate during scratching but not swimming; and flexion reflex-selective neurons increase their spike rate during flexion reflex but not swimming or scratching.

### Electrophysiological analysis

Mean action potential parameters were measured while each neuron fired at a low rate. Spike height was measured from resting potential to peak and afterhyperpolarization (AHP) amplitude from resting potential to trough. Spike width was measured at action potential threshold. AHP duration was measured between crossing below resting potential until the half-maximal AHP amplitude.

Dual-referent phase histograms of intracellularly recorded neuron spike rates were calculated with respect to the onset and offset of bursts of a nerve, usually HF but in some cases HE, during swimming and scratching motor patterns, using a custom script written in Spike2 (Cambridge Electronic Design). In dual-referent phase histograms, each cycle is divided into nerve-active and nerve-inactive phases (Orlovsky, 1972; Burns and Usherwood, 1979; Drew and Doucet, 1991; Berkowitz and Stein, 1994b; Westberg et al., 1998; Tresch and Kiehn, 1999; Berkowitz, 2001a). If the signal for HF nerve activity was too low, HE was used for this purpose instead. Phase histogram data were then used to calculate the mean vector (Mardia, 1972; Batschelet, 1981; Drew and Doucet, 1991; Berkowitz and Stein, 1994b). The mean vector length (MVL) indicates the degree of rhythmic modulation; an MVL of 0 would indicate no phase preference with respect to the hip nerve activity cycle, whereas an MVL of 1 would indicate that all action potentials occurred in the same phase (1/10) of each cycle. The mean vector angle (MVA) indicates the neuron's spiking phase preference within the HF cycle; an MVA of 0–180° (or 0–0.5) would indicate a phase preference during the HF bursts, and an MVA of 180–360° (or 0.5–1.0) would indicate a phase preference within the HF interburst intervals. The null hypothesis that the neuron's firing occurred at random with respect to the nerve activity cycle was evaluated using the Rayleigh test (Mardia, 1972; Batschelet, 1981; Drew and Doucet, 1991; Berkowitz and Stein, 1994b). Neurons were not used to calculate the mean vector if they fired fewer than 10 action potentials during a motor pattern. MVAs were used in analyses only if the phase histogram passed the Rayleigh test with *p* < 0.05. The scratch MVL used for comparison to the swim MVL was the average of the MVLs for all available forms of scratching. The scratch MVA used for comparison with the swim MVA was for the form of scratching that passed the Rayleigh test with the lowest *p* value, unless phase histograms for multiple forms of scratching had *p* < 0.001, in which case the one among these with the highest MVL was used (Berkowitz and Stein, 1994b; Berkowitz, 2001a, 2002, 2008). Cycles of scratching motor patterns that showed HE deletions (Robertson et al., 1985) were excluded from analyses of MVL and MVA, oscillation amplitudes, and oscillation peak and trough phases to improve consistency.

### Membrane potential oscillations

Following the calculation of spike rate phase histograms, action potentials (identified by threshold crossings) were deleted, with the voltage values on either side interpolated, in Spike2; multiple cycles of membrane potentials were averaged to create a dual-referent phase–averaged membrane potential, from which the oscillation amplitude, peak phase, and trough phase were measured (Berkowitz, 2005, 2008; Bannatyne et al., 2020). If the peak and trough phases were calculated with respect to the onset and offset of HE instead of HF, then 0.5 was added to or subtracted from these values to assess all peak and trough phases with respect to the HF activity cycle (Bannatyne et al., 2020). If more than one form of scratching (rostral, pocket, caudal) was evoked, then the motor pattern with the highest oscillation amplitude was used for comparisons of oscillation amplitude, peak phase, and trough phase for scratching. In some cases, 1.0 (=360°) was added to or subtracted from the peak or trough phase for display purposes only (Fig. 8); note that adding or subtracting 1.0 does not change phase preference and that possible correlations between cyclical variables were assessed via circular, not linear, statistical tests.

### Statistics

For single, pairwise comparisons of linear variables, we used the Mann–Whitney nonparametric test to assess statistical significance. We used the two-tailed Spearman's rank–rank correlation test to assess correlations (*r*_s_) between pairs of linear variables—spike parameters, oscillation amplitudes, and MVLs—across neurons. For comparisons of linear variables across more than two groups, we used the Kruskal–Wallis nonparametric test followed by Dunn's post hoc test for pairwise comparisons. We used the *χ*^2^ test to assess differences in proportions. We assessed correlations between cyclical or phase variables—MVAs, peak phases, and trough phases—using the circular–circular correlation test (Batschelet, 1981; Berkowitz, 2008) to calculate the circular–circular correlation coefficient, *r*_c_. Note that the circular–circular correlation test of paired angular values requires that one first assesses whether each set of values is uniformly distributed throughout the cycle. If both sets are uniformly distributed (i.e., *p* > 0.05 for each set in the Rayleigh test), then the circular–circular correlation coefficient and the probability are calculated; if *p* < 0.05 in this second step, then there is a statistically significant correlation within pairs. If one or both sets are not uniformly distributed (i.e., they do pass the Rayleigh test with *p* < 0.05), however, then one cannot continue to the second step and cannot conclude that there is a statistically significant correlation of values within pairs. We calculated mean angular or phase differences between pairs of angles or phases as the MVA of the phase difference within each pair, provided that there was a significant correlation, *r*_c_, between these cyclical variables.

## Results

**Figure 1.**
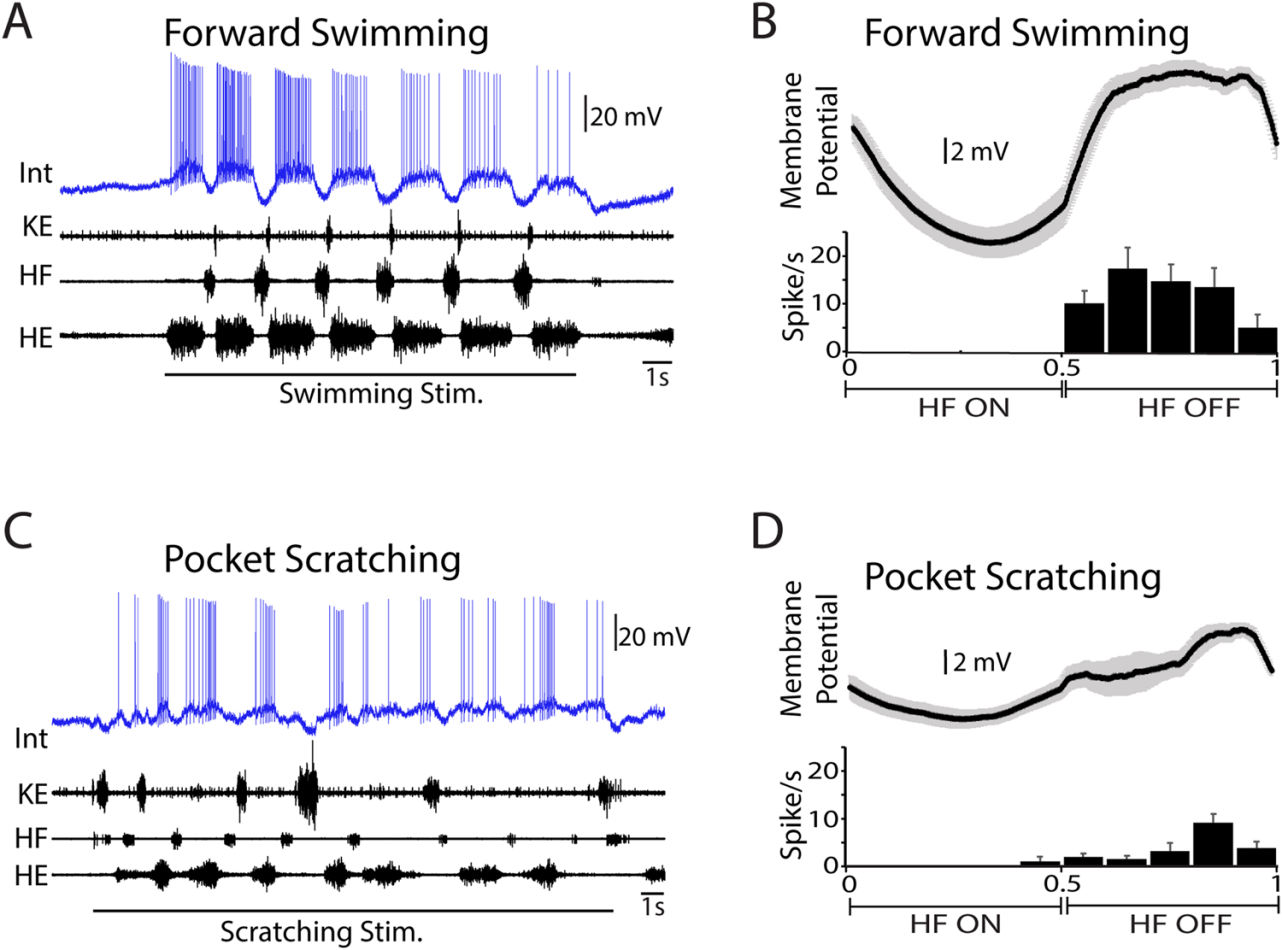
Example of a multifunctional neuron, activated during both swimming and scratching. ***A***, Activation during swimming. ***B***, Dual-referent spike rate phase histogram (below) and phase-averaged membrane potential (above) during swimming (5 cycles). ***C***, Activation during pocket scratching. ***D***, Dual-referent spike rate phase histogram and phase-averaged membrane potential during pocket scratching (8 cycles). Int, intracellular recording; KE, knee-extensor nerve; HF, hip-flexor nerve; HE, hip-extensor nerve; error bars and gray shading, SEM.

**Figure 2.**
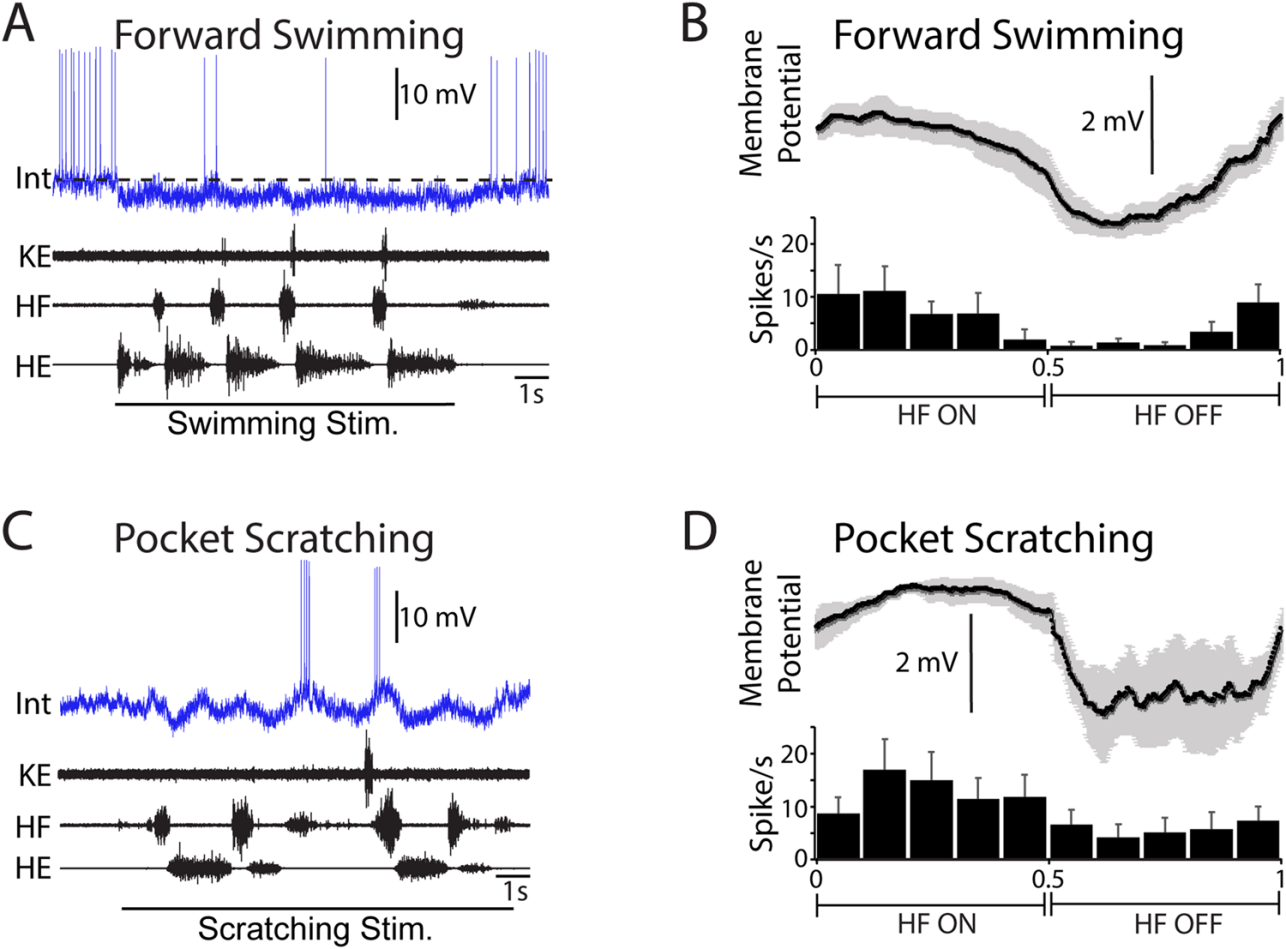
Example of a scratch-specialized neuron, activated during scratching but not swimming. ***A***, Inhibition during swimming; the horizontal dashed line indicates baseline membrane potential prior to motor pattern. ***B***, Spike rate phase histogram and phase-averaged membrane potential during swimming (7 cycles). ***C***, Activation during pocket scratching. ***D***, Spike rate phase histogram and phase-averaged membrane potential during pocket scratching (10 cycles). Int, intracellular recording; KE, knee-extensor nerve; HF, hip-flexor nerve; HE, hip-extensor nerve; error bars and gray shading, SEM.

Sixty-eight neurons activated during swimming, scratching, and/or flexion reflex were analyzed, including multifunctional neurons, scratch-specialized neurons, flexion reflex-selective neurons, and a swim-specialized neuron. All 68 were studied during both swimming and scratching; 62 of the 68 were also studied during flexion reflex. Each multifunctional neuron (Fig. 1*A*–*D*) by definition increases its mean spike rate during both swimming (Fig. 1*A*) and scratching (Fig. 1*C*; Berkowitz, 2002). The spike rate phase histograms and phase-averaged membrane potentials for the neuron shown in Figure 1 showed strong rhythmic modulation during both swimming (Fig. 1*B*; MVL, 0.75; *p* < 0.001; MVA, 0.73) and scratching (Fig. 1*D*; MVL, 0.71; *p* < 0.001; MVA, 0.81). This neuron was recorded from the D8 segment at a depth of 910 µm, likely in the dorsal half of the ventral horn. Each scratch-specialized neuron (Fig. 2*A*–*D*) by definition increases its mean spike rate during scratching (Fig. 2*C*), but not during swimming (Fig. 2*A*; Berkowitz, 2002). The neuron shown in Figure 2 was hyperpolarized during swimming, evidenced by the membrane potential dropping below its baseline level (indicated by a dashed line in Fig. 2*A*) during swimming. Its spike rate phase histograms and phase-averaged membrane potentials showed rhythmic modulation during both swimming (Fig. 2*B*; MVL, 0.51; *p* < 0.001; MVA, 0.12) and scratching (Fig. 2*D*; MVL, 0.29; *p* < 0.001; MVA, 0.24), but this rhythmic modulation was weaker than that shown by the multifunctional neuron in Figure 1, *B* and *D*. This neuron was recorded from the D10 segment at a depth of 880 µm, also likely in the dorsal half of the ventral horn.

**Figure 3.**
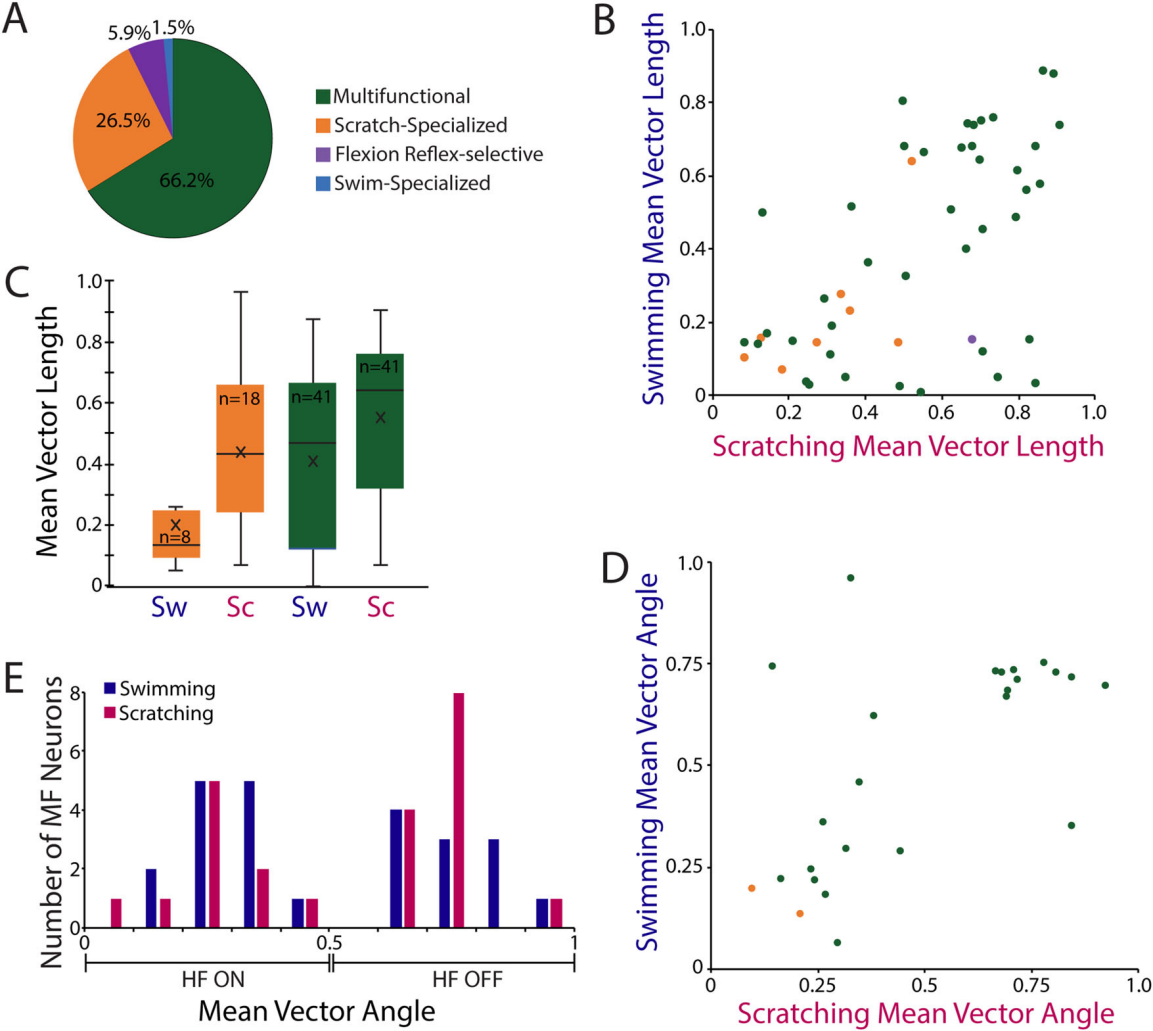
MVLs and MVAs for neuron types. ***A***, Proportions of neuron types recorded. ***B***, A scatterplot of swimming versus scratching MVLs for multifunctional neurons and scratch-specialized neurons. ***C***, A box-and-whisker plot of swimming and scratching MVLs for multifunctional neurons and scratch-specialized neurons; thick bars indicate middle quartiles, x indicates mean, and horizontal line indicates median. ***D***, A scatterplot of MVAs for multifunctional and scratch-specialized neurons with statistically significant MVLs. ***E***, Histogram of MVAs during swimming and scratching for multifunctional neurons with statistically significant MVLs. Sw, swimming; Sc, scratching; MF, multifunctional; HF, hip flexor; *p < 0.05.

We recorded from 45 multifunctional neurons, 18 scratch-specialized neurons, four flexion reflex-selective neurons, and one swim-specialized neuron (Fig. 3*A*). Thus, multifunctional neurons were more frequently encountered than behaviorally specialized neurons.

Although scratch-specialized neurons by definition do not increase their mean firing rate during swimming, some still fired enough action potentials during swimming for the mean vector to be calculated (see Materials and Methods). An MVL of 1.0 would indicate the greatest possible rhythmicity of firing, while an MVL of 0 would indicate the least possible rhythmicity (see Materials and Methods). For neurons overall that spiked sufficiently during both swimming and scratching to calculate the MVLs for both, neurons that had highly rhythmic spiking (i.e., had a high MVL) during one motor pattern (swimming or scratching) tended to have highly rhythmic spiking during both motor patterns, while those that were relatively nonrhythmic for one motor pattern were relatively nonrhythmic for both (Fig. 3*B*; *r*_s _= 0.51; *p* = 0.00014; Spearman's test). The mean and median MVLs were substantially higher for scratching and swimming for multifunctional neurons as a group than for scratch-specialized neurons as a group (Fig. 3*C*) and also higher for each of these groups during scratching than during swimming (Fig. 3*C*). The MVLs for this set of four categories showed statistically significant differences by the nonparametric Kruskal–Wallis test (*p* = 0.004). Post hoc comparisons via the nonparametric Dunn's test, however, did not show any statistically significant pairwise differences (*p* > 0.05). Thirty-five (78%) of the 45 multifunctional neurons and 15 (83%) of the 18 scratch-specialized neurons had significantly rhythmic firing (Rayleigh test *p* < 0.05; see Materials and Methods) during scratching. Twenty-six (58%) of the 45 multifunctional neurons but only two (11%) of the 18 scratch-specialized neurons had significantly rhythmic spiking during swimming.

**Figure 4.**
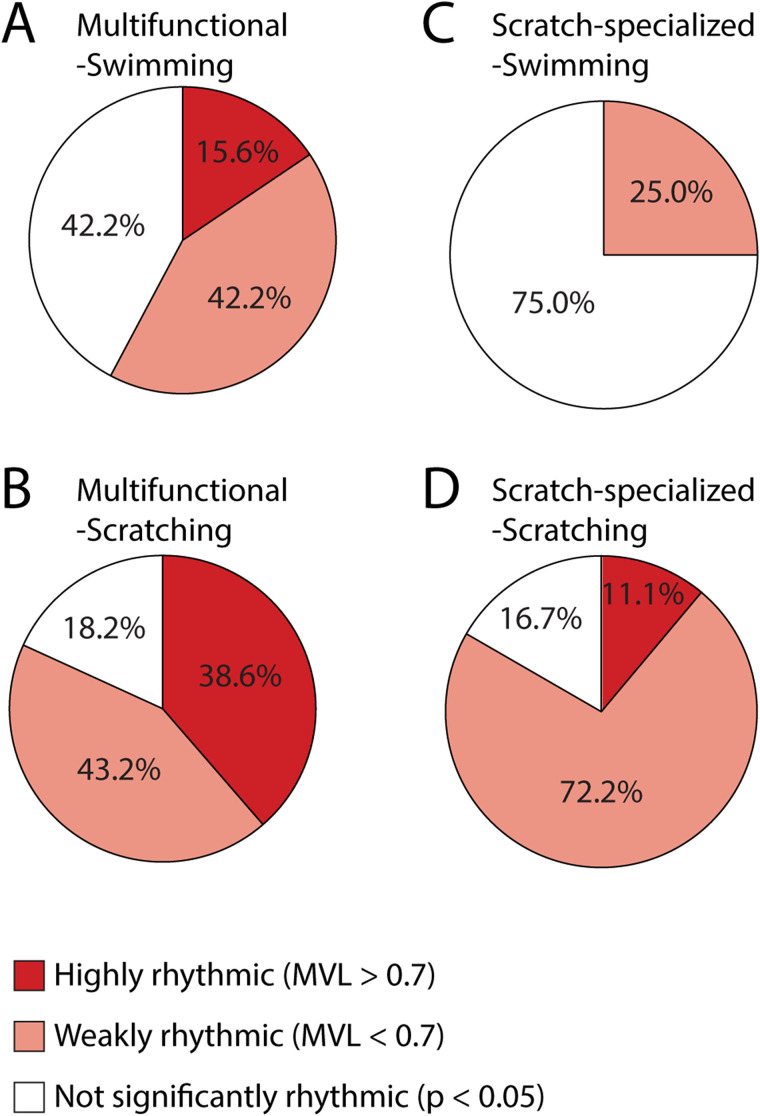
Proportions of highly rhythmic, weakly rhythmic, and not significantly rhythmic multifunctional neurons (***A***, ***B***; *n* = 45) and scratch-specialized neurons (***C***, ***D***; *n *= 18) during swimming (***A***, ***C***) and scratching (***B***, ***D***).

Also, if one defines highly rhythmic neurons as those with MVL > 0.7, 16% of all multifunctional neurons for which the MVL could be calculated were highly rhythmic during swimming (Fig. 4*A*), and 39% were highly rhythmic during scratching (Fig. 4*B*), while none of the scratch-specialized neurons were highly rhythmic during swimming (Fig. 4*C*) and only 11% were highly rhythmic during scratching (Fig. 4*D*). The proportions of neurons that were highly rhythmic (Fig. 1), weakly rhythmic (Fig. 2), and nonrhythmic were significantly different for multifunctional versus scratch-specialized neurons during both swimming and scratching (*p* < 0.0001 for each; *χ*^2^ test). Thus, the analyses of spike rate phase histograms suggest that a higher proportion of multifunctional neurons than scratch-specialized neurons are highly rhythmic.

Analysis of the MVAs for neurons that had significantly rhythmic spiking (see Materials and Methods) for both swimming and scratching indicates that nearly all rhythmic neurons had similar phase preferences within the HF activity cycle across motor patterns (Fig. 3*D*). MVAs were significantly correlated between swimming and scratching for all neurons together (circular–circular correlation test, *r*_c _= 0.61; *p* < 0.001). The mean phase difference (MVA) between the swimming and scratching MVAs was 0.04, indicating that phase preferences within the HF cycle were nearly the same during swimming and scratching. Within multifunctional neurons (which made up 23 of the 25 neurons for which MVA could be assessed), swimming MVAs were also highly correlated with scratching MVAs (*r*_c _= 0.59; *p* < 0.001), and the mean phase difference between them was 0.04, indicating that they had essentially the same spiking phase preference within the HF activity cycle for swimming and scratching. Multifunctional neurons appeared to be bimodal in their firing phase preferences, with two peaks, at 0.2–0.4 and 0.6–0.8 (Fig. 3*E*). This could indicate that multifunctional neurons comprise two groups, one most active during HF-on and another during HF-off.

**Figure 5.**
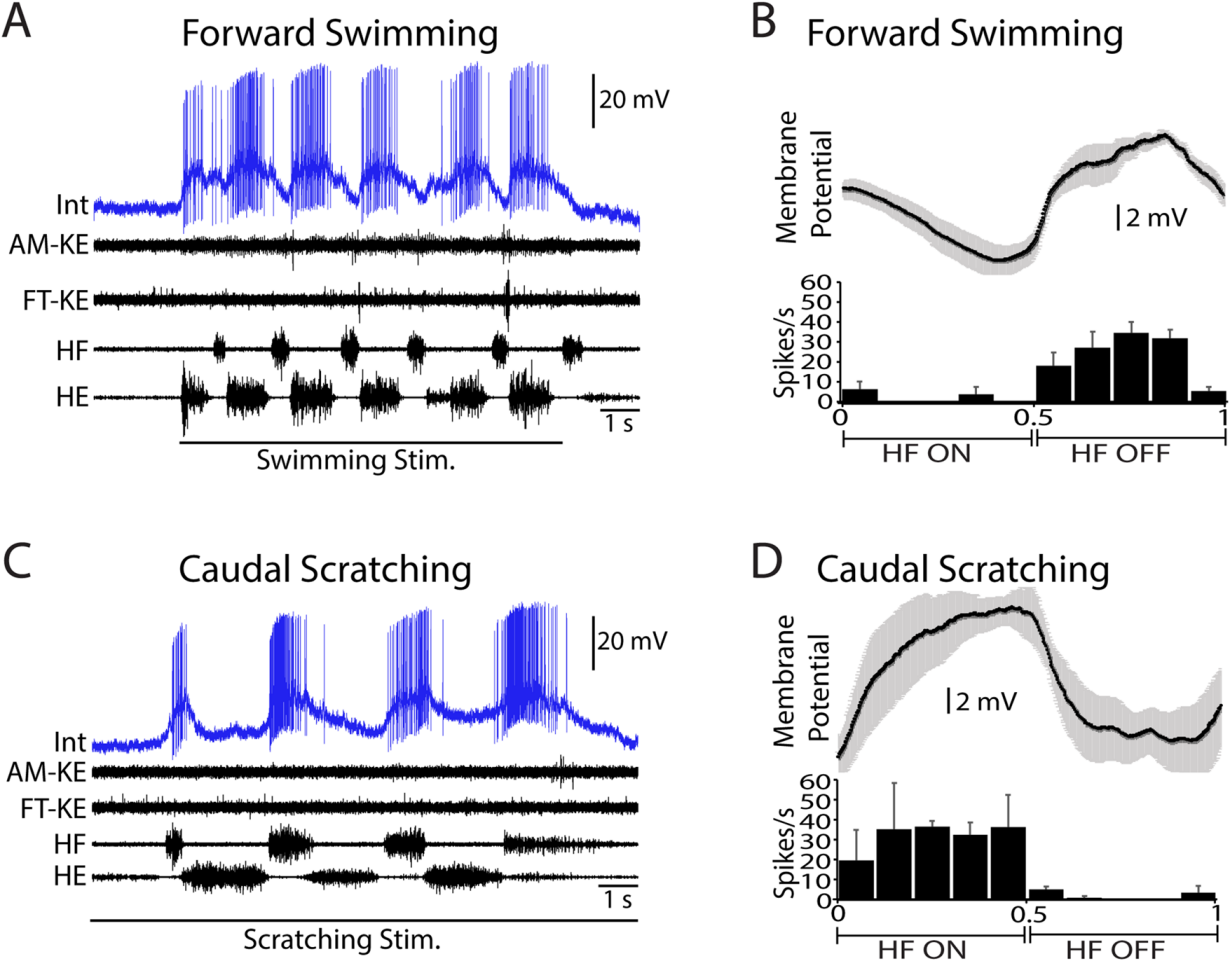
A multifunctional neuron that switched phase preferences within the hip activity cycle between swimming and scratching. ***A***, Rhythmic activation during HE phase of swimming. ***B***, Spike rate phase histogram and phase-averaged membrane potential during swimming (4 cycles). ***C***, Rhythmic activation during HF phase of caudal scratching. ***D***, Spike rate phase histogram and phase-averaged membrane potential during caudal scratching (3 cycles). Intracellular recordings were low-pass filtered (cutoff, 370 Hz) for display purposes. Int, intracellular recording; AM-KE, ambiens knee-extensor nerve; FT-KE, femorotibialis knee-extensor nerve; HF, hip-flexor nerve; HE, hip-extensor nerve; error bars and gray shading, SEM.

Although neurons overall tended to have the same phase preferences within the HF cycle for swimming and scratching, they could have opposite phase preferences, as shown in Figure 5. This neuron fired almost exclusively during HF interburst intervals (i.e., during the HE bursts) in forward swimming (Fig. 5*A*,*B*; MVL, 0.68; *p* < 0.001; MVA, 0.74) but almost exclusively during HF bursts in caudal scratching (Fig. 5*C*,*D*; MVL, 0.64; *p *< 0.001; MVA, 0.28). In all cases, it tended to fire when the knee extensors were not firing (Fig. 5*A*,*C*). This neuron was recorded in the S1 segment at a depth of 980 µm, likely in the dorsal half of the ventral horn.

Eight (20%) of the 41 multifunctional neurons tested and 8 (50%) of the 16 scratch-specialized neurons tested also increased their firing rate during flexion reflex. Of these neurons also having statistically significant MVAs, three (60%) of the five multifunctional and four (57%) of the seven scratch-specialized neurons had a firing phase preference during the HF-on phase of scratching (0–0.5), while four (80%) of the five multifunctional neurons and the one scratch-specialized neuron had a phase preference during the HF-on phase of swimming.

**Figure 6.**
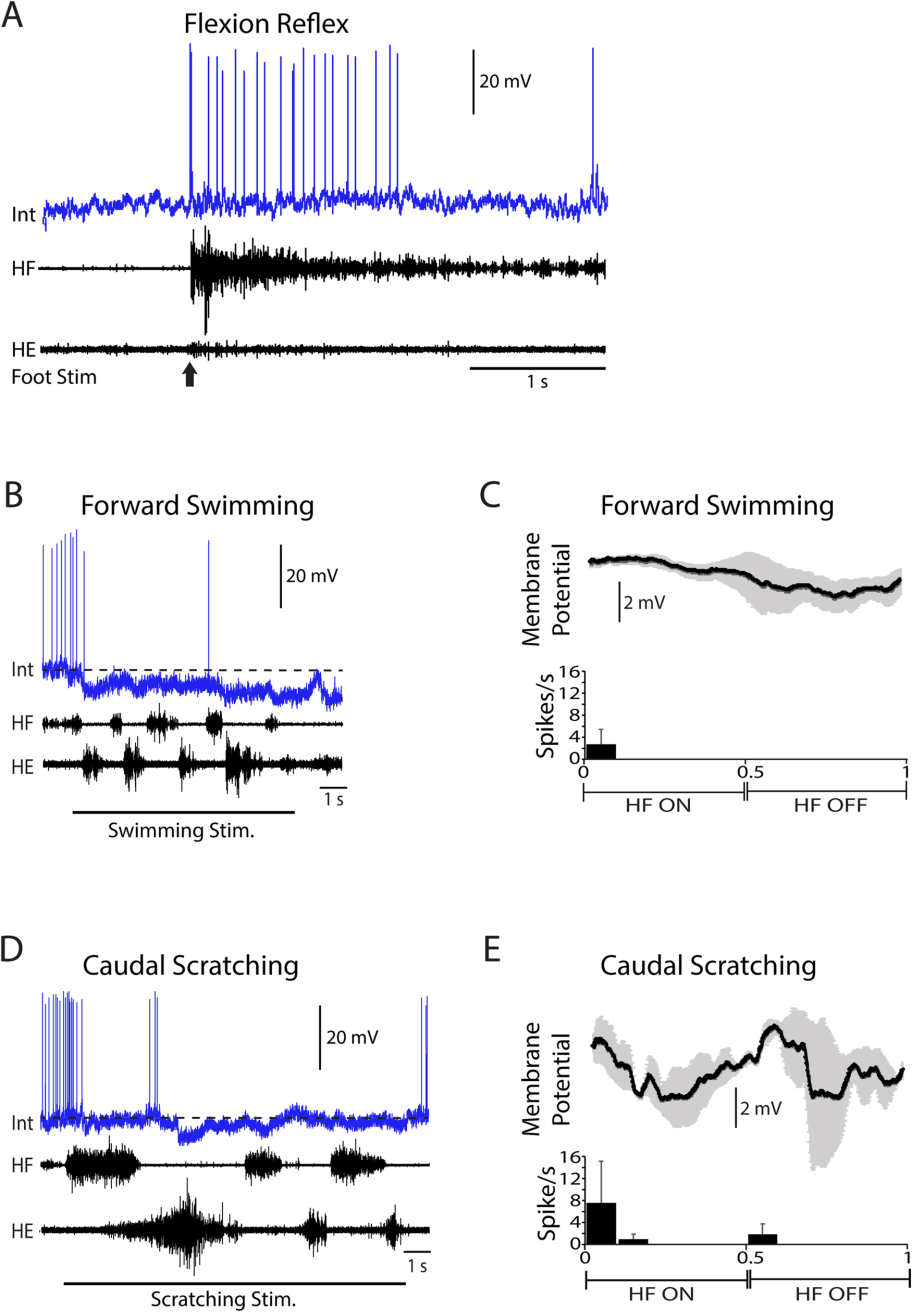
Example of a flexion reflex-selective neuron. ***A***, Activation during flexion reflex. ***B***, Inhibition during swimming. ***C***, Spike rate phase histogram and phase-averaged membrane potential during swimming (3 cycles). ***D***, Inhibition during caudal scratching. ***E***, Spike rate phase histogram and phase-averaged membrane potential during caudal scratching (2 cycles). Horizontal dashed lines in ***B*** and ***D*** indicate baseline membrane potential prior to motor pattern. Int, intracellular recording; KE, knee-extensor nerve; HF, hip-flexor nerve; HE, hip-extensor nerve; error bars and gray shading, SEM.

Flexion reflex-selective neurons increase their firing rate during flexion reflex, but not during scratching or swimming (Fig. 6*A*,*B*,*D*). The flexion reflex-selective neuron shown in Figure 6, like most, was hyperpolarized during scratching and swimming (Fig. 6*B*,*D*), evidenced by the membrane potential dropping below the baseline (Fig. 6*B,D*, dashed lines). This neuron displayed only a weak rhythmic oscillation during both swimming and scratching [Fig. 6*C*,*E*; swimming, <10 spikes (see Materials and Methods); scratching, MVL, 0.64; *p* = 0.015; MVA, 0.06]. This neuron was recorded in the D7 segment at a depth of 540 µm, likely in the deep dorsal horn or the intermediate zone.

Mean resting membrane potentials, action potential heights and widths, and AHP amplitudes and durations were calculated (Table 1) for multifunctional neurons, scratch-specialized neurons, flexion reflex-selective neurons, and the swim-specialized neuron. None of these parameters differed significantly between multifunctional and scratch-specialized neurons (Mann–Whitney tests; *p* > 0.5 for each comparison; flexion reflex-selective and swim-specialized neurons not tested due to low sample size).

**Table 1.** Means ± standard deviations of neuron and spike parameters

Neuron type	*V_m_* (mV)	Spike amp (mV)	Spike width (ms)	AHP amp (mV)	AHP dur (ms)
All neurons	−61.0 ± 13.2	54.8 ± 14.0	2.2 ± 0.9	8.5 ± 5.0	7.7 ± 13.6
Multifunctional	−61.3 ± 14.0	54.7 ± 14.8	2.1 ± 0.9	8.5 ± 4.7	7.3 ± 15.3
Scratch-specialized	−62.4 ± 12.3	53.1 ± 12.4	2.4 ± 1.1	8.4 ± 5.6	7.9 ± 9.8
Flexion reflex-selective	−57.6 ± 9.9	44.4 ± 11.8	1.5 ± 0.5	6.8 ± 4.7	10.0 ± 12.3
Swim-specialized	−46.4	60.0	2.7	16.3	9.5

*V_m_*, membrane potential; amp, amplitude; dur, duration.

**Figure 7.**
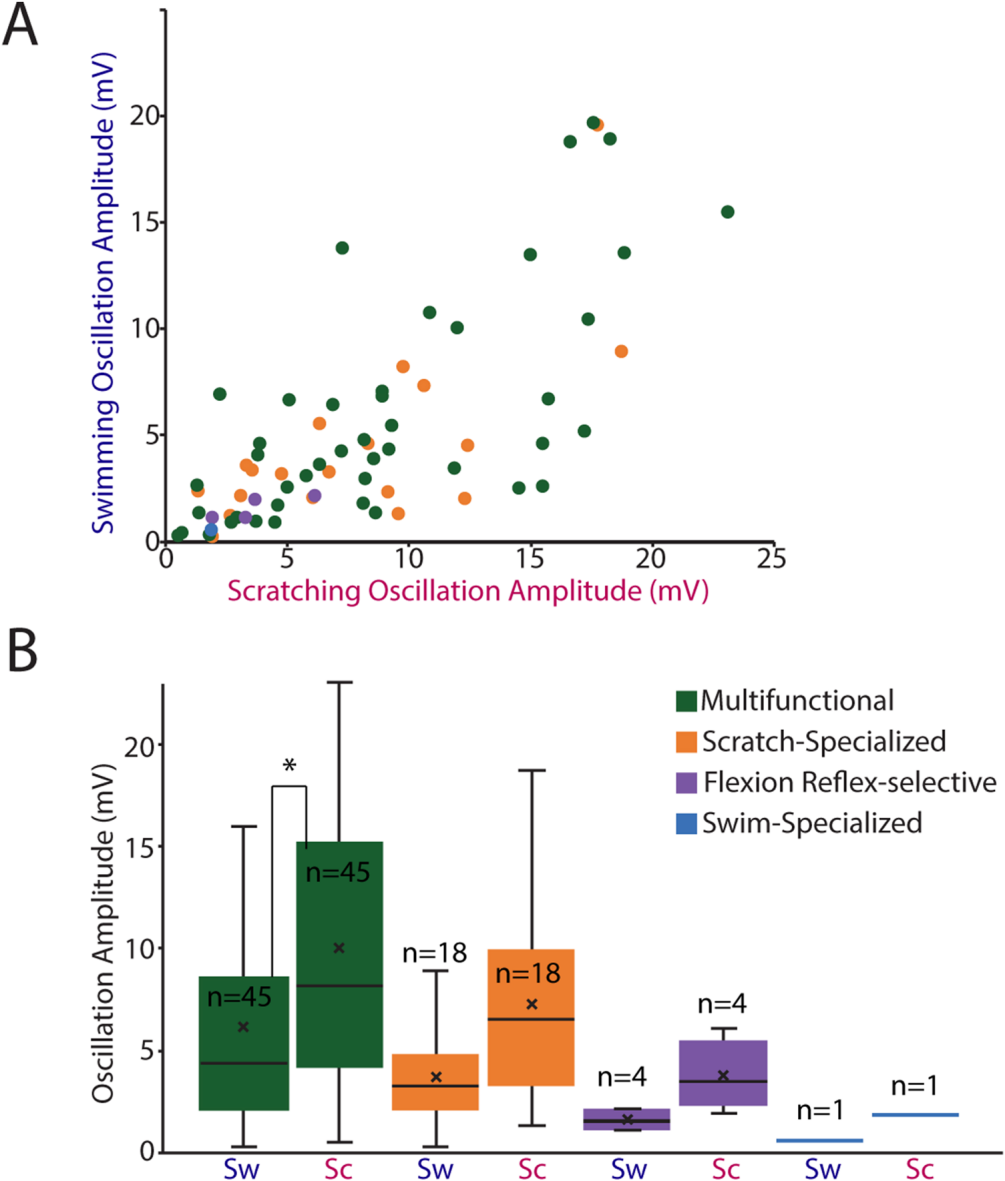
Phase-averaged membrane potential oscillation amplitudes. ***A***, A scatterplot of all neurons by type; one outlier is not displayed. ***B***, A box-and-whisker plot of neurons by type during both swimming (Sw) and scratching (Sc); thick bars indicate middle quartiles, x indicates mean, and horizontal line indicates median; *p < 0.05 by Dunn's test.

Phase-averaged membrane potential oscillations allow for analysis of rhythmicity of multifunctional and specialized neurons during swimming and scratching, whether or not they fired action potentials, and thus facilitate analysis of a larger set of intracellularly recorded neurons, as well as assessment of correlations between spike parameters and membrane potential oscillation parameters. Neurons with large oscillations during one motor pattern (swimming or scratching) tended to have large oscillations during both (Fig. 7*A*), echoing the findings for MVLs. Swimming and scratching oscillation amplitudes were correlated for neurons overall (*r*_s _= 0.72; *p* = 0.000001; Spearman's test) and were significantly greater during scratching than swimming (*p* = 0.0003; Mann–Whitney test). Oscillation amplitudes showed significant variation across two neuron types (multifunctional and scratch-specialized) and the two motor pattern types (swimming and scratching; *p* = 0.0016, Kruskal–Wallis test). Oscillation amplitude means and medians (Fig. 7*B*) were larger during scratching than during swimming for both multifunctional neurons and scratch-specialized neurons, again echoing the findings for MVLs. Oscillation amplitude means and medians were also higher for multifunctional than for scratch-specialized neurons, during both swimming and scratching (Fig. 7*A*,*B*), also like the MVL findings. Using Dunn's nonparametric post hoc test, one pairwise comparison showed a statistically significant effect: multifunctional neurons had a higher oscillation amplitude during scratching than during swimming (*p* < 0.05). Flexion reflex-selective neurons had relatively small oscillation amplitudes during both swimming and scratching (Fig. 7*A*,*B*), compared with both multifunctional and scratch-specialized neurons; statistical comparisons could not be made because there were only four flexion reflex-selective neurons.

Analysis of the consistency of peak and trough phases of phase-averaged membrane potentials during swimming and scratching may help determine whether excitation or inhibition plays a greater role in creating rhythmic modulation. We used the circular–circular correlation test to assess possible correlations between pairs of phases for peak–trough, peak–peak, and trough–trough comparisons during swimming and scratching; when there was a statistically significant correlation, we calculated the mean phase difference between the pairs of phases as the MVA of all the phase differences (see Materials and Methods).

**Figure 8.**
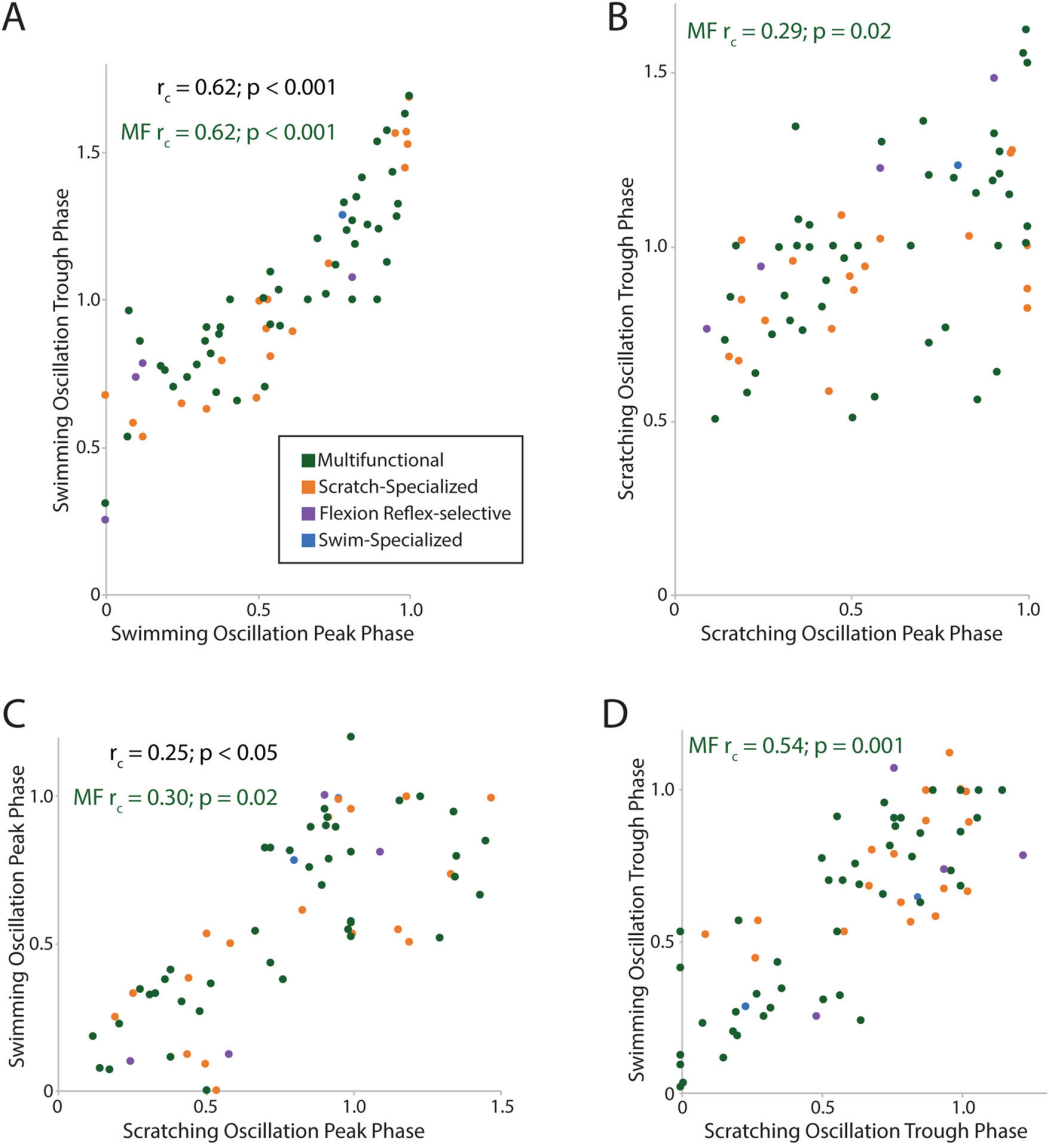
Comparisons of membrane potential oscillation peak and trough phases across behaviors and types of neuron. ***A***, Swimming peak versus swimming trough. ***B***, Scratching peak versus scratching trough. ***C***, Swimming peak versus scratching peak. ***D***, Swimming trough versus scratching trough.

For all neurons together, peak and trough phases were significantly correlated with each other during swimming (Fig. 8*A*; *r*_c _= 0.62; *p* < 0.001), but this could not be assessed for scratching (Fig. 8*B*), as the trough distribution was not uniform (see Materials and Methods). The mean difference between the peak and trough phases (i.e., the MVA of the phase differences) was 0.45 for swimming, indicating that the peak and trough were approximately out of phase (=0.5) during swimming. For multifunctional neurons, peak and trough phases were significantly correlated with each other during both swimming (Fig. 8*A*; *r*_c _= 0.62; *p* < 0.001) and scratching (Fig. 8*B*; *r*_c _= 0.29; *p* = 0.02). The mean difference between the peak and trough phases of multifunctional neurons was 0.45 for swimming and 0.50 for scratching, indicating that the peak and trough were approximately out of phase in multifunctional neurons during both swimming and scratching.

For all neurons together, swimming peak phases were significantly correlated with scratching peak phases (Fig. 8*C*; *r*_c _= 0.25; *p* < 0.05), but trough phases (Fig. 8*D*) could not be assessed, as the scratching distribution was nonuniform (see Materials and Methods). The mean difference between their swimming and scratching peak phases was 0.08, indicating that neurons had similar peak phases during these two rhythmic motor patterns. For multifunctional neurons, swimming and scratching peak phases were significantly correlated with each other (*r*_c _= 0.30; *p* = 0.02), and swimming and scratching trough phases were also correlated with each other (*r*_c _= 0.54; *p* < 0.001). The mean difference between the swimming and scratching peak phases of multifunctional neurons was 0.06, and the mean difference in the trough phases was 0.02, indicating that each neuron had approximately the same peak phase and the same trough phase during swimming compared with scratching. Collectively, these results suggest that multifunctional neurons receive both rhythmic excitation and rhythmic inhibition, but rhythmic inhibition (which may create the oscillation troughs) likely plays a greater role than rhythmic excitation in the rhythmic modulation of multifunctional neurons during both swimming and scratching, because the trough phases were more consistent.

Flexion reflex involves primarily hip flexion, so one might expect that neurons activated during flexion reflex would be most depolarized during the HF bursts of swimming and scratching, but this was not always the case. Of the multifunctional and scratch-specialized neurons that increased their firing rate during flexion reflex, six (75%) of the eight multifunctional neurons and five (71%) of the seven scratch-specialized neurons had an oscillation peak during the HF-on phase of scratching, while five (63%) of the eight multifunctional neurons and two (25%) of the eight scratch-specialized neurons had an oscillation peak during the HF-on phase of swimming.

We tested for correlations between spike parameters and linear parameters that indicate rhythmicity—MVL and oscillation amplitude—during both swimming and scratching, for neurons overall (Table 2). The only significant correlations were between spike height and oscillation amplitude and between AHP duration and oscillation amplitude, in each case for both swimming and scratching oscillations. AHP duration was negatively correlated with oscillation amplitude, i.e., neurons with briefer AHPs had larger oscillations.

**Table 2.** Spearman rank–rank correlations between spike parameters and measures of rhythmicity during swimming and scratching

	Sw Osc Amp *r_s_*	Sw Osc Amp *p*	Sc Osc Amp *r_s_*	Sc Osc Amp *p*	Sw MVL *r_s_*	Sw MVL *p*	Sc MVL *r_s_*	Sc MVL *p*
Membrane potential	−0.21	0.15	−0.27	0.057	−0.15	0.36	−0.15	0.31
Spike height	0.32	0.007	0.31	0.01	0.1	0.46	0.03	0.79
Spike width	0.08	0.54	0.006	0.97	0.14	0.33	0.006	0.96
AHP amplitude	0.08	0.50	0.17	0.17	0.11	0.43	0.12	0.32
AHP duration	−0.36	0.002	−0.26	0.03	−0.07	0.62	−0.08	0.51

Sw, swimming; Sc, scratching; Osc, oscillation; Amp, amplitude; MVL, mean vector length; *r*_s_, correlation coefficient; *p*, probability; AHP, afterhyperpolarization. Note that the only statistically significant correlations were between spike height and oscillation amplitudes and between AHP duration and oscillation amplitudes.

**Figure 9.**
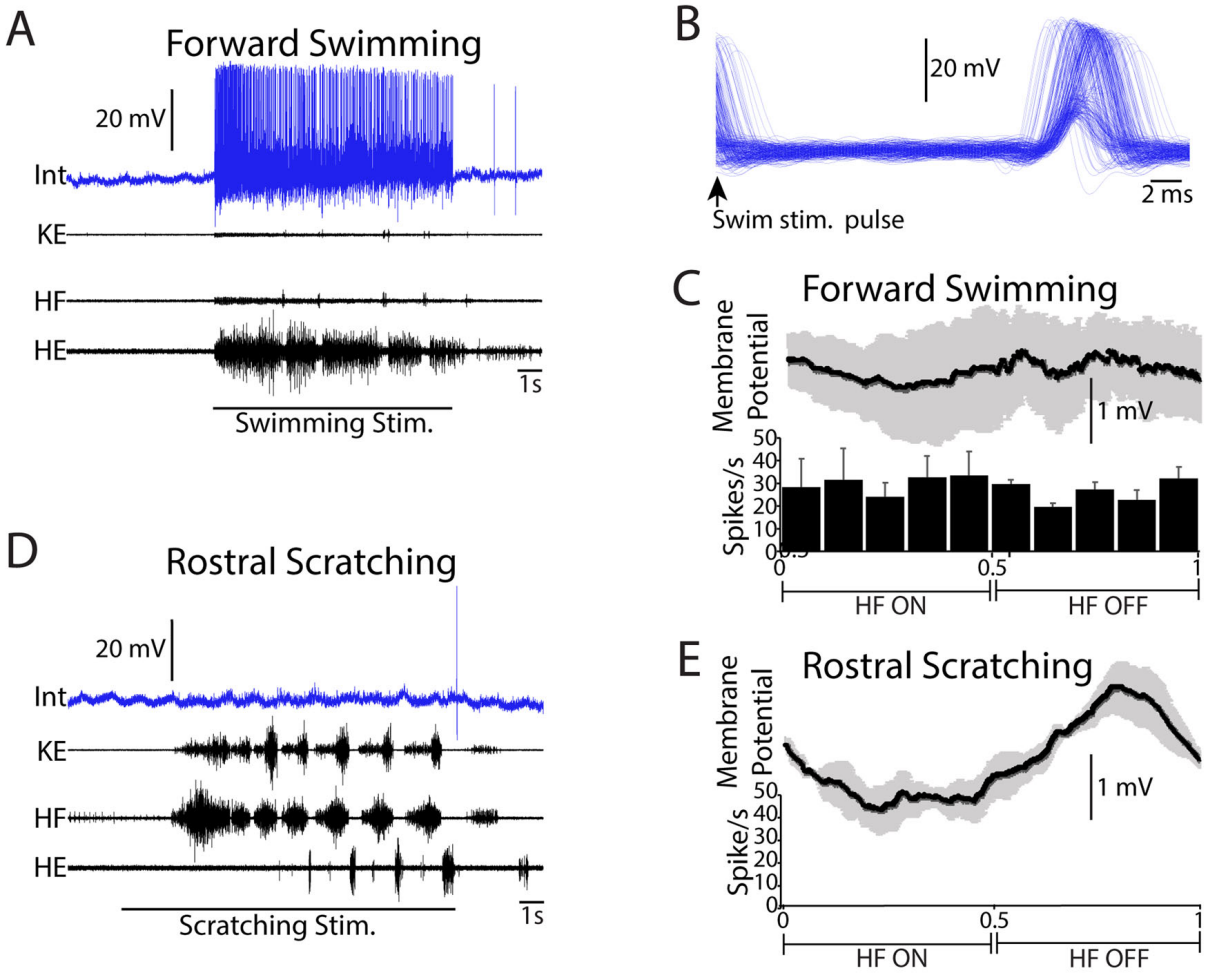
Swim-specialized neuron. ***A***, Tonic activity during swimming. ***B***, Superimposed responses to successive swim-stimulation pulses. ***C***, Spike rate phase histogram and phase-averaged membrane potential during swimming (3 cycles). ***D***, No spiking during rostral scratching. ***E***, Phase-averaged membrane potential during scratching (4 cycles). Intracellular recordings were low-pass filtered (cutoff: 220 Hz) for display. Int, intracellular recording; KE, knee-extensor nerve; HF, hip-flexor nerve; HE, hip-extensor nerve; error bars and gray shading, SEM.

We have previously recorded intracellularly from scratch-specialized and flexion reflex-selective neurons, but never from a turtle swim-specialized neuron. In the current dataset, we found one swim-specialized neuron, recorded in the D9 segment at a depth of 490 µm, likely in the deep dorsal horn (Fig. 9). This neuron was tonically activated during swimming without any apparent rhythmic oscillations (Fig. 9*A*,*C*; MVL, 0.03; *p* = 0.73). When superimposing the neuron's responses to swim-stimulation pulses, the neuron displayed a consistent excitatory postsynaptic potential of ∼15 mV and usually an action potential, at a variable latency of ∼20 ms (Fig. 9*B*); this suggests that this neuron was excited oligosynaptically by one or more of the contralateral dorsolateral funiculus (cDLF) axons that we stimulated to trigger swimming. It was not active during rostral scratching but fired once just after scratch stimulation ended, likely via postinhibitory rebound (Fig. 9*D*). The neuron had a small rhythmic membrane potential oscillation during rostral scratching, with a peak during HF-off (Fig. 9*E*). The neuron did not respond during flexion reflex (data not shown).

**Figure 10.**
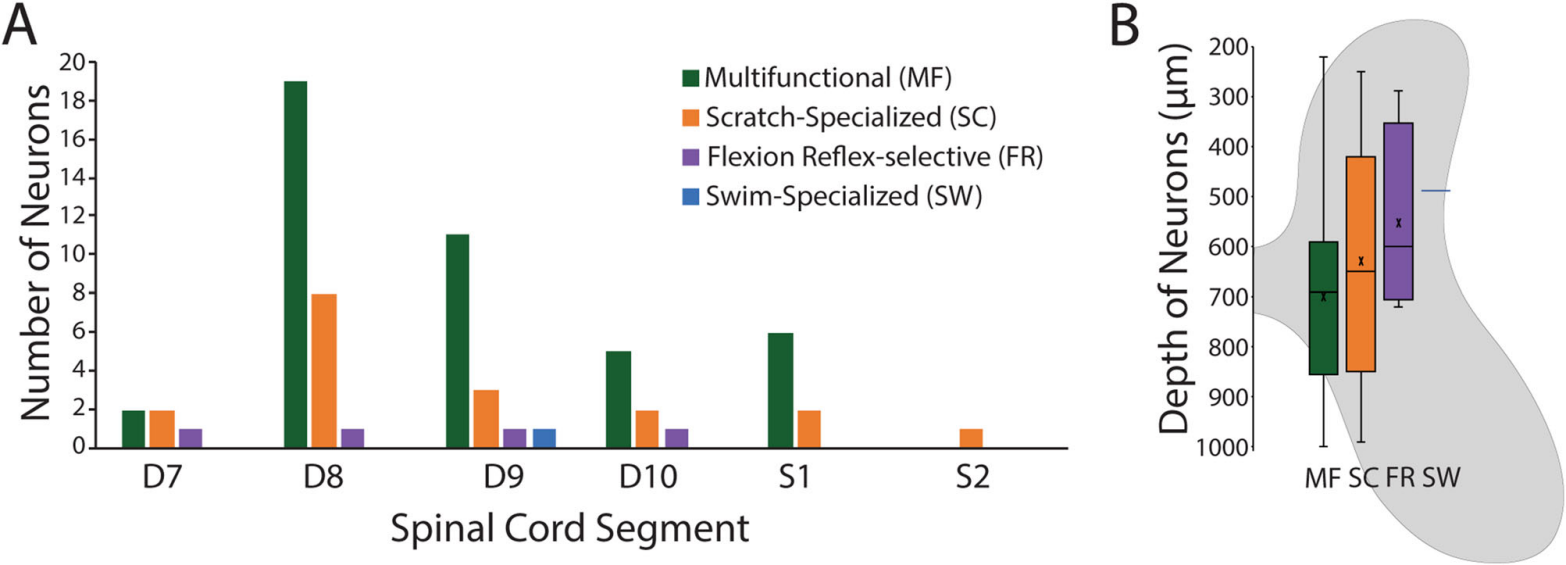
Locations of each type of neuron recorded. ***A***, Locations by spinal segments. ***B***, A box-and-whisker plot of locations by distance from spinal cord surface; thick bars indicate middle quartiles, x indicates mean, and horizontal line indicates median. Graph is overlain on a schematic spinal cord cross section to illustrate approximate gray-matter dorsoventral locations.

With the exception of the one swim-specialized neuron, neurons of each type could be found throughout the spinal segments examined (Fig. 10*A*). As a group, flexion–reflex-selective neurons and the one swim-specialized neuron tended to be more dorsal than most multifunctional neurons, while scratch-specialized neurons were widely spread dorsoventrally (Fig. 10*B*), but these differences were not statistically significant (*p* = 0.30; Kruskal–Wallis test).

## Discussion

This study demonstrates that spinal neurons tend to have a similar degree of rhythmic modulation of their spike rate during swimming and scratching. In addition, multifunctional neurons (activated during both swimming and scratching) tend to have more rhythmic firing than behaviorally specialized neurons (scratch-specialized neurons and flexion reflex-selective neurons), and a much larger subset of multifunctional neurons than specialized neurons had highly rhythmic firing. These findings are consistent with previous studies of turtle multifunctional neurons using extracellular single-neuron (Berkowitz, 2002) and intracellular recording (Berkowitz, 2008; Bannatyne et al., 2020), but the current study included more neurons than the previous two intracellular studies combined. Evidence from this study together with previous intracellular studies collectively also suggests that neurons tend to have similar oscillation amplitudes during swimming and scratching. In addition, oscillation amplitudes tend to be larger in multifunctional neurons than in scratch-specialized, flexion reflex-selective, or swim-specialized neurons (Berkowitz, 2007, 2008; Bannatyne et al., 2020). Collectively, these findings suggest that a subset of multifunctional neurons with large oscillations and highly rhythmic firing are likely part of the CPG for both swimming and scratching. Multifunctional neurons also tended to be more ventrally located. Neurons that are more rhythmic were also previously found to be more ventrally located in the turtle spinal cord (Berkowitz, 2001a).

**Figure 11.**
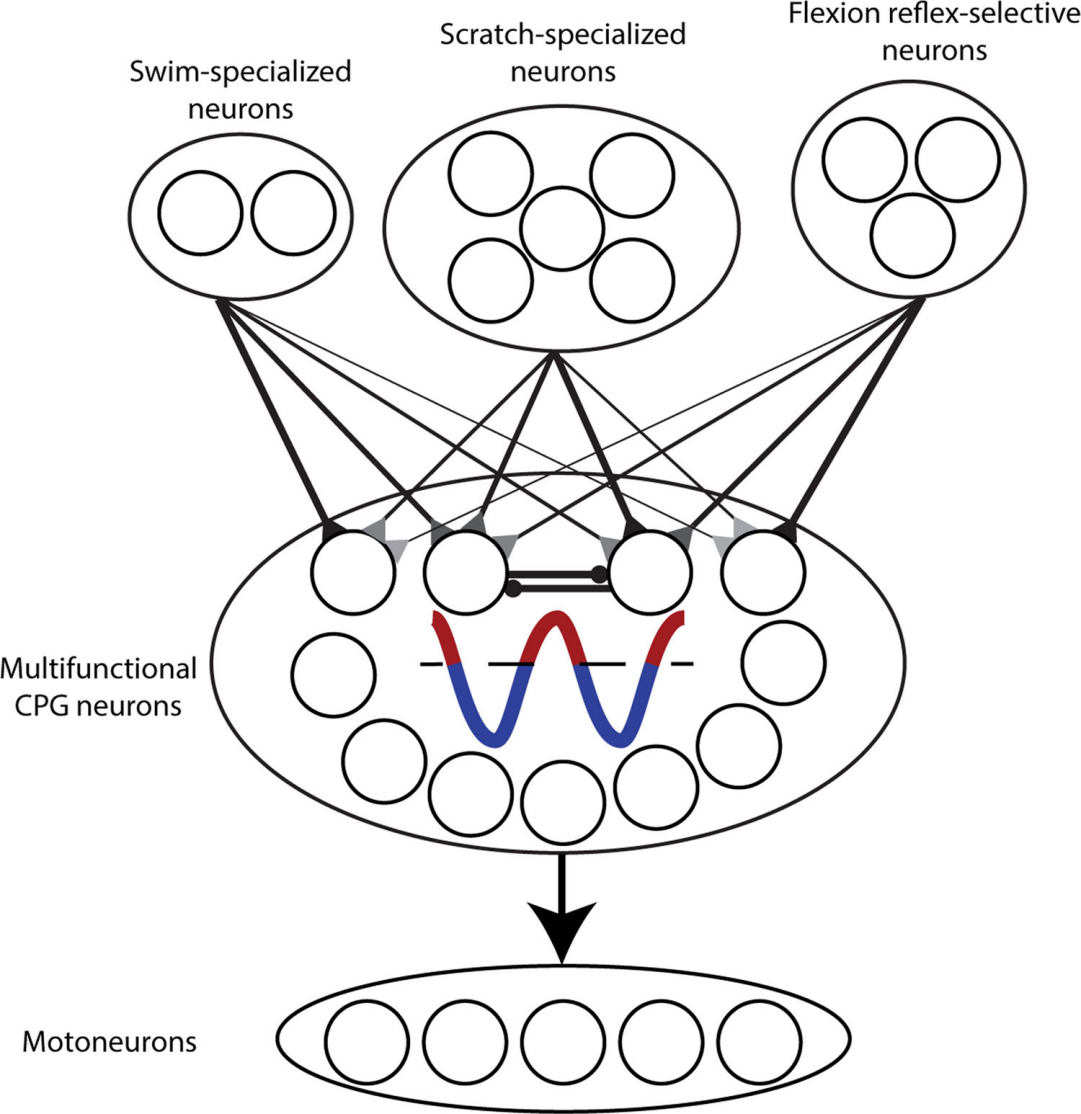
Schematic diagram to illustrate main findings: CPG neurons are likely a subset of multifunctional neurons, as more multifunctional neurons are highly rhythmic. The rhythmicity of multifunctional neurons is mostly due to rhythmic inhibition. Behaviorally specialized neurons likely trigger specific motor patterns via differential activation of multifunctional CPG neurons.

Previous turtle intracellular studies involving fewer neurons found that the trough phases of rhythmic oscillations were correlated between swimming and scratching in multifunctional neurons, while the peak phases were not (Berkowitz, 2008; Bannatyne et al., 2020). In the larger dataset of the current study, we found that both the peak phases and the trough phases were significantly correlated between swimming and scratching, but the trough phases were more highly correlated than the peak phases. This suggests that multifunctional neurons receive both rhythmic excitation and rhythmic inhibition, but rhythmic inhibition likely plays a greater role than rhythmic excitation. A previous extracellular recording study found that neurons were more rhythmic when they fired at lower frequency (Berkowitz and Stein, 1994b), which is also consistent with rhythmic inhibition playing a key role in creating rhythmicity. Recently, immunocytochemistry has suggested that most of these multifunctional neurons are themselves inhibitory (Bannatyne et al., 2020), so it is likely that many CPG neurons are both rhythmically inhibited and inhibitory. Thus, multifunctional interneurons may create the HF–HE alternation largely via rhythmic inhibition of each other, during both swimming and scratching motor patterns (Fig. 11). Multifunctional neurons also tend to be rhythmic and inhibitory in tadpole and larval zebrafish swimming and struggling, involving left–right axial alternation (Berkowitz et al., 2010), which is analogous to HF–HE alternation in the turtle.

We also found that multifunctional neurons tended to have similar phase preferences within the HF activity cycle during swimming and scratching, whether assessed by spike rate phase histograms (MVAs) or membrane potential oscillation peak phases, consistent with previous studies (Berkowitz, 2002, 2008; Bannatyne et al., 2020), but using a larger dataset than previous intracellular studies. We found that the phase preferences (i.e., MVAs) of multifunctional neurons tended to cluster in the middle of the HF-on phase or the middle of the HF-off phase. A similar bimodal distribution of phase preferences can be seen in one previous turtle study (Berkowitz and Stein, 1994b; Fig. 5*A*), while other previous turtle studies seemed to show a continuous variation in phase preferences (Berkowitz, 2001a, 2002; Linden et al., 2022). This discrepancy might be accounted for if most rhythmic neurons control the timing of HFs or HEs, while smaller numbers of other neurons control the timing of muscles that act on other joints or on multiple joints, which themselves typically burst in different phases of the hip activity cycle during different behaviors. We did find one neuron in this study that fired almost exclusively during opposite phases of the HF activity cycle during swimming and scratching, in both cases at the time that the knee extensors we recorded were not firing. Thus, this neuron might either have inhibited knee-extensor or excited knee-flexor motoneurons. Rhythmic turtle interneurons dedicated to knee timing have been noted previously (Berkowitz, 2001a; Stein and Daniels-McQueen, 2003) but seem to be much less common than neurons controlling hip timing.

Fewer scratch-specialized neurons than multifunctional neurons were highly rhythmic during swimming and/or scratching, consistent with previous studies (Berkowitz, 2002, 2008). Thus, most scratch-specialized neurons are unlikely to be CPG neurons. Flexion reflex-selective neurons we studied here had even smaller rhythmic oscillations during swimming and scratching than scratch-specialized neurons, consistent with a previous finding (Berkowitz, 2007). Thus, we suggest that scratch-specialized and flexion reflex-selective neurons are involved in triggering scratching and flexion reflex, respectively, rather than generating motor output more directly. The more dorsal locations of flexion reflex-selective neurons, here and in a previous study (Berkowitz, 2007), are consistent with this idea. Thus, behaviorally specialized neurons might bias a multifunctional CPG toward a particular motor pattern, e.g., by activating HF-exciting CPG neurons (or inhibiting HE-exciting CPG neurons) to help trigger scratching or flexion reflex instead of swimming (Fig. 11).

Neurons with greater spike heights tended to have larger oscillations during both swimming and scratching. This might have occurred simply because the driving force was greater in these neurons. More intriguingly, neurons with briefer AHPs tended to have larger oscillations during both swimming and scratching. We previously found that transverse interneurons (T neurons), which we define morphologically, tended to have both briefer AHPs and larger oscillations than non-T neurons, making them good candidates to contribute to rhythm generation (Berkowitz et al., 2006). Most if not all T neurons are multifunctional, so they may be a key contributors to rhythm generation for both swimming and scratching (Berkowitz, 2008). The correlations found in the current study are consistent with this hypothesis.

This is the first study to record intracellularly from a turtle swim-specialized neuron, to the best of our knowledge. This neuron, likely in the deep dorsal horn, was tonically excited with no apparent rhythmic modulation of its membrane potential during swimming and did not spike during scratching or flexion reflex. Thus, this neuron might have activated the swimming CPG, without contributing to generation of the swimming rhythm. Following each swim-stimulation pulse (to the D3 cDLF), this neuron displayed an excitatory postsynaptic potential at a latency of ∼20 ms, plus one action potential following most pulses, consistent with this neuron acting as an intermediary between (likely reticulospinal; Lennard and Stein, 1977) axons being stimulated to trigger swimming and the swimming CPG neurons. Swim-specialized neurons have also been observed many other species, including hatchling tadpoles, larval zebrafish, and leeches (Briggman et al., 2005; Berkowitz et al., 2010). The relative rarity of turtle swim-specialized neurons is consistent with the idea, previously suggested for leeches, that swimming evolved later (in this case, compared with scratching) and mainly made use of existing neural circuitry (Briggman and Kristan, 2006).

## Synthesis

Reviewing Editor: Silvia Pagliardini, University of Alberta Decisions are customarily a result of the Reviewing Editor and the peer reviewers coming together and discussing their recommendations until a consensus is reached. When revisions are invited, a fact-based synthesis statement explaining their decision and outlining what is needed to prepare a revision will be listed below. The following reviewer(s) agreed to reveal their identity: NONE. Note: If this manuscript was transferred from JNeurosci and a decision was made to accept the manuscript without peer review, a brief statement to this effect will instead be what is listed below.

The authors are appreciated for considering the suggestions and revising the draft accordingly. However, there are still a couple of major points that need to be addressed.

1. As previously noted by myself and another reviewer, the categorisation of neurons is questionable and requires reconsideration. Figure 2 illustrates the interneuron's involvement in both behaviours, as evidenced by the equal averaged firing rates and mean Vm. While I appreciate the clarification on the classification done in here, I believe there may be better ways to classify neurons or even opt out from doing it, despite previous usage of this method.

To further support the argument, the authors defined 'scratch-specialised' neurons are highly engaged during swimming, as shown in Figure 7. The amplitude of Vm oscillation in scratch-specialised neurons is similar to that in multifunctional neurons during swimming. This demonstrates that both groups of neurons are similarly engaged during swimming, and therefore the term 'scratch-specialised' is misleading.

Furthermore, the present categorisation heavily relies on the neuron's resting state, that is, its firing activity before the behaviour is initiated. For instance, if a neuron is already spiking before the motor behaviour is induced, the average spiking rate may not increase significantly due to strong inhibition during the behaviour. Conversely, if the same neuron is not firing before the motor behaviour, it is more likely to exhibit an increased firing rate. In conclusion, the current classification is highly dependent on the resting state. Since the provided examples demonstrate significant changes in the resting state between behaviours, the categorisation is not reliable.

In conclusion, it is highly recommended to conduct the analysis of the data without pre-defined categories of neurons. This is supported by the pooled data (Figure 3 and 7), which indicates a continuum of parameters with both groups intermixed, rather than clear 'classes' of interneurons.

2. Intrinsic properties of neurons. It is understandable that the experiments have already been performed and there is "no way to back". However, the existing data can be better utilised. From the recordings, it is possible to obtain resting Vm and AP properties such as height, duration, and AHP, and examine how they relate to patterns of activity during behaviour.

Minor point.

Since turtle spinal cord is not a commonly studied preparation, it may be difficult to fully appreciate the depth of the recorded neuron depicted in Figure 10. To improve clarity, it is suggested to include an example transverse cut of the spinal cord (or a schematic) to indicate the location of the recorded neurons.

SD or SEM could be plotted as shadows in line-plots. This is a routine practice and is not "distracting" or "too difficult to see".
